# Robust Adaptive Recurrent Cerebellar Model Neural Network for Non-linear System Based on GPSO

**DOI:** 10.3389/fnins.2019.00390

**Published:** 2019-05-29

**Authors:** Jian-sheng Guan, Shao-jiang Hong, Shao-bo Kang, Yong Zeng, Yuan Sun, Chih-Min Lin

**Affiliations:** ^1^College of Electrical Engineering and Automation, Xiamen University of Technology, Xiamen, China; ^2^Princess Margaret Cancer Centre, University Health Network, Toronto, ON, Canada; ^3^Department of Electrical Engineering, Innovation Center for Big Data and Digital Convergence, Yuan Ze University, Taoyuan, Taiwan

**Keywords:** RARC neural network, GPSO algorithm, learning rate, robot manipulator system, non-linear systems

## Abstract

A robust adaptive recurrent cerebellar model articulation controller (RARC) neural network for non-linear systems using the genetic particle swarm optimization (GPSO) algorithm is presented in this study. The RARC is used as the principal tracking controller and the robust compensation controller is designed to recover the residual of the approximation error. In the RARC neural network, the steepest descent gradient method and the Lyapunov function are used for deriving the adaptive law parameter of the system. Besides, the learning rates play an important role in these adaptive laws and they have a great effect on the functions of control systems. In this paper, the combination of the genetic algorithm with the mutation particle swarm optimization algorithm is applied to seek for the optimal learning rates of the RARC adaptation laws. The numerical simulations about the inverted pendulum system as well as the robot manipulator system are given to confirm the effectiveness and practicability of the GPSO-RARC-based control system. Compared with other control schemes, the proposed control scheme is testified to be reliable and can obtain the optimal parameter about the learning rates and the minimum root mean square error for non-linear systems.

## Introduction

Strictly speaking, almost all practical control systems are non-linear systems and there is a difference between the mathematical model and the practical system. Besides, the structure and parameters of the practical systems are generally unknown or time-varying and the disturbances acting on the system are often random and unmeasurable in many cases. The neural network has the advantages of highly parallel structure, powerful learning ability, continuous non-linear function approximation ability, fault tolerance, etc., which greatly promotes and expands the application of neural network technology in non-linear system identification and control (Hunt et al., 1992). Recently, scholars have proposed generous research papers on neural network control theory and engineering application. For a class of uncertain non-linear systems with strict feedback, an adaptive neural network controller was designed using dynamic surface control technique (Wang and Huang, 2005). For a class of uncertain Multiple-Input Multiple-Output non-linear systems with unknown control coefficient matrix and input non-linearity, a variable structure control method combining an adaptive neural network controller with backtracking and Lyapunov synthesis is proposed (Chen et al., 2010). The paper presented a control scheme for the non-linear systems with input and state delay, which merges a radial basis function neural network, backstepping, and adaptive control (Zhu et al., 2008). As for a class of second-order non-linear systems, a wavelet adaptive backstepping control system was designed, which consists of a neural backstepping controller and a robust controller (Hsu et al., 2006).

In 1975, Albus proposed the concept of the cerebellar model articulation controller (CMAC) for the first time (Albus, 1975), which was an imitation of the cerebellum learning structure, also one of the local approximations in the neural network system. Cerebellar model network system not only has non-linear approximation ability, adaptive generalization ability and associative memory ability, but also is a kind of fast convergence neural network, which has been widely used in non-linear real-time control system (Guan et al., 2016). An efficient controller was proposed for the robot manipulators based on the structure and local learning characteristics of CMAC (Commuri et al., 1997). Considering the characteristics of non-linear uncertainty model, the paper presented an adaptive connection controller based on the monitoring system, which was composed of a supervisory controller and adaptive CMAC (Lin and Peng, 2004). Compared with fully connected neural networks, the CMAC neural network (NN) has strong structural advantages and was an effective control method for unknown dynamics non-linear systems (Commuri and Lewis, 1995). For a class of multi-input and multi-output uncertain non-linear systems, a self-organizing CMAC control system was proposed, which combines sliding mode control, compensation control and CMAC (Lin and Chen, 2009). At the same time, a TKS fuzzy CMAC controller integrates the robust compensation and adaptive law is proposed to improve the precision of position control and speed control for robot manipulators (Guan et al., 2018).

As a kind of artificial neural network, recurrent neural network takes sequence data as input, recursion in the direction of sequence evolution and all nodes are linked in a chain to form a closed loop. Therefore, it can show dynamic time series behavior. Unlike feed forward neural networks, RNNs have the characteristics of memory and parameter sharing. This performance also makes it extremely useful for speech recognition, language modeling, machine translation and other fields (Sak et al., 2014). A complex fuzzy neural network system, which can be a modified version of the fuzzy neural networks, was used for identifying and controlling non-linear dynamic systems (Zhong et al., 2017, 2018; Lam, 2018). The recursive neuron has an internal feedback loop which can capture the dynamic response of the system and further simplify the network model (Lee and Teng, 2000). The paper combines Takagi-Sugeno-Kang fuzzy model with the wavelet neural network and constructs a recurrent wavelet fuzzy neural network to identify and predict the operation of non-linear dynamic systems (Lin and Chin, 2004). For the non-linear uncertain systems, an adaptive recurrent CMAC with sliding mode control was proposed, and the performance of the system was proved on the car-following system and the chaotic system (Lin and Chen, 2006). For the motion control of the linear ultrasonic motor, an adaptive recurrent CMAC based on variable optimal learning rate and dynamic gradient descent method was studied (Peng and Lin, 2007).

Particle swarm optimization (PSO) algorithm is an evolutionary algorithm proposed by Kennedy and Eberhart in 1995 (Eberhart and Kennedy, 1995). It is derived from the simulation of bird predation and is an evolutionary computation technology based on swarm intelligence. As a new parallel optimization evolutionary algorithm, PSO can deal with a large number of non-linear, non-differentiable, multi-peak as well as non-continuous optimization and multi-peak optimization problems, which was widely used in engineering and science fields (Kennedy, 2011). In the 1970s, professor J. H. Holland first developed the model of Genetic algorithm (GA) (Holland, 1973). It is an effective optimization method with principles about genetics natural selection. GA is also very popular in the fields of optimal scheduling, computer science, combinatorial optimization as well as transportation problem since its simple and universal, strong robustness and parallel processing (Holland, 1992). The improvement of genetic algorithm in the past is generally considered as the problem of premature and convergence. An adaptive genetic algorithm with dynamic fitness function for multi-objective problems in a dynamic environment was proposed to review the performance of the algorithm (Bingul, 2007). A new kind of genetic algorithms combined with the concept of the horizontal set was proposed to control the “precocity” of the genetic algorithm (Qinghua et al., 2006). In order to improve the convergence of the genetic algorithm, an improved crossover operation is proposed, and new population diversity and individual correlation are defined (Cai and Xia, 2006). However, the research on genetic algorithm fails to fully consider the situation of individuals in each generation, which does not match the growth and improvement of individuals in the process of evolution. In the selection and cross steps of the genetic algorithm, individuals directly enter the next generation, while individuals themselves do not get improved. Individuals have to grow and adapt to the environment in order to reproduce in nature. In the PSO algorithm, each particle is related to each other, and the particle can be imitated in the natural world, and the maximum performance can be improved and the particle can be mature (Peng et al., 2008).

There are usually two options to select the appropriate learning rate of the recurrent CMAC (learning rate of the recurrent neuron, weight, the variance and the mean), one of which is to adopt human expert experience. However, the accuracy of the method is not high, and not suitable for complex and uncertain problems. The second scheme is gradient learning (Song et al., 2008; Misra and Saha, 2010). In this paper, a robust adaptive recurrent cerebellar model neural network for non-linear system based on GPSO algorithm is investigated, in order to avoid trial-and-error and improve the local optimal problems. In this system, the optimal learning rate for controller is calculated by GPSO algorithm, the adaptive recurrent cerebellar model articulation controller is used as the principal tracking controller and the robust compensation controller is designed to recover the residual of the approximation error, and the steepest descent gradient method and the Lyapunov function are used for deriving the online adaptive law parameter, so that the system stability can be guaranteed. Finally, the proposed GPSO-RARC-based control system is applied to the inverted pendulum system and the robot manipulator system to illustrate its effectiveness. Compared with the existing research already reported in the literature, the contribution of this paper has the following three aspects: (1) This paper combines genetic algorithm and mutation particle swarm optimization algorithm to find the optimal learning rate of adaptive law to the robust adaptive recurrent cerebellar model articulation controller and reduce the system training time; (2) The proposed control scheme ensures the stability of the entire system; (3) The compensation control can eliminate the small disturbance, when there are uncertainty, the compensation control deals with the lumped uncertainty. The full text is structured as follows. After a basic introduction, the formulation of the non-linear control system is shown in section Problem Formulation. In section GPSO-RARC, a GPSO-RARC control system is developed. Section Simulation Results provides the simulation results about the manipulator system and the inverted pendulum system. Finally, in Section Conclusion some valuable conclusions are drawn from the results.

## Problem Formulation

The *n*th order non-linear system can be denoted as:

(1) $$\begin{array}{l} \{\begin{array}{l} x^{\left(n\right)}(t)=f(\underline{x}(t))+g(\underline{x}(t))u(t)+d(t)\\ y=x(t) \end{array} \end{array}$$

or, equivalent to formulas

(2) $$\begin{array}{l} \{\begin{array}{l} {\dot{x}}_{1}=x_{2}\\ {\dot{x}}_{2}=x_{3}\\ \;\vdots \\ {\dot{x}}_{n}=f(x_{1},x_{2},\cdots ,x_{n})+g(x_{1},x_{2},\cdots ,x_{n})u(t)+d(t)\\ y=x_{1} \end{array} \end{array}$$

in which *f*(***x***(*t*)) ∈ **ℜ**^*m*^ and *g*(***x***(*t*)) ∈ **ℜ**^*m*×*m*^ represent smooth non-linear uncertain functions, which are assumed to be bounded, but functions that are assumed to be bounded, and assume *g*(***x***(*t*)) is invertible; $u\left(t\right)={\;\left[\;u_{1}\left(t\right),u_{2}\left(t\right),\cdots ,u_{m}\left(t\right)\;\right]\;}^{T}\in {ℜ}^{m}$ and $x\left(t\right)={\;\left[\;x_{1}\left(t\right),x_{2}\left(t\right),\cdots ,x_{m}\left(t\right)\;\right]\;}^{T}\in {ℜ}^{m}$ are the inputs and outputs of the control, respectively; $\underline{x}(t)={\left[x^{T}(t),\;{\dot{x}}^{T}(t),\;\cdots ,\;x^{\left(n-1\right)}{\;}^{T}(t)\right]}^{T}\in {ℜ}^{mn}$ is a state vector of the system and is assumed to be measurable, and $d\left(t\right)={\;\left[\;d_{1}\left(t\right),d_{2}\left(t\right),\cdots ,d_{m}\left(t\right)\;\right]\;}^{T}\in {ℜ}^{m}$ is the unknown external disturbance but bounded (|*d*(*t*)| ≤ *D*).

The purpose of the control system is to design a controller so that the state ***x***(*t*) can track a given reference value ***x***_*d*_(*t*). The tracking error was denoted as $e(t)\underline{\underline{\Delta }}\;x_{d}(t)-x(t)\in {ℜ}^{m}$, and the tracking error vector of the control system is defined as:

(3) $$\begin{array}{l} E\underline{\underline{\Delta }}\;{\left[e^{T}(t),{\dot{e}}^{T}(t),\cdots ,e^{(n-1)T}(t)\right]}^{T}\in {ℜ}^{mm} \end{array}$$

If the dynamics and external disturbances of the controlled object are known (i.e., the nominal functions of *f*(***x***(*t*)), *g*(***x***(*t*)) and ***d***(*t*) are known exactly), the so-called feedback linearization method can be used for the control problem. In this way, an ideal controller can be developed as:

(4) $$\begin{array}{l} u^{*}=\frac{1}{g(\underline{x}(t))}\left[-f(\underline{x}(t))-d(t)+x_{d}{\;}^{\left(n\right)}+{\underline{K}}^{T}E\right] \end{array}$$

in which $\underline{K}={\left[K_{n},\cdots ,K_{2},K_{1}\right]}^{T}\in {ℜ}^{mn\times m}$ is the feedback gain matrix, where ${\text{K}}_{i}=diag\left(k_{i1},\;k_{i2},\;\cdots \;,\;k_{im}\right)\in {ℜ}^{m\times m}$ (*i* = 1, 2, ⋯ , *n*) are non-zero positive constants diagonal matrix. The following error dynamics are derived by applying the control law (4) to the system (1). Suppose ***K*** is chosen so that all roots of the polynomial $h(s)\;\underline{\underline{\Delta }}\;s^{n}+k_{1}s^{n-1}+\cdots +k_{n}$ are strictly in the open left half of the complex plane. This means that for any starting initial conditions, the trace of the reference trajectory is asymptotically achieved at $\underset{t\to \infty }{\lim}|E|=0$.

Substituting (4) into (1) the error dynamic equation is developed as:

(5) $$\begin{array}{l} e^{\left(n\right)}+K_{1}e^{\left(n-1\right)}+\cdots +K_{n}e=e^{\left(n\right)}+{\underline{K}}^{T}E=0 \end{array}$$

However, the non-linear functions *f*(***x***) and *g*(***x***) are usually unknown and external disturbances are unknown and uncertain. In this case, the control law (4) cannot be implemented in the practical applications. In order to make the system output ***x***(*t*) effectively follow the given reference track ***x***_*d*_(*t*), a GPSO-RARC control system is developed to achieve a better control performance in the following sections.

## GPSO-RARC

**Figure 1:**
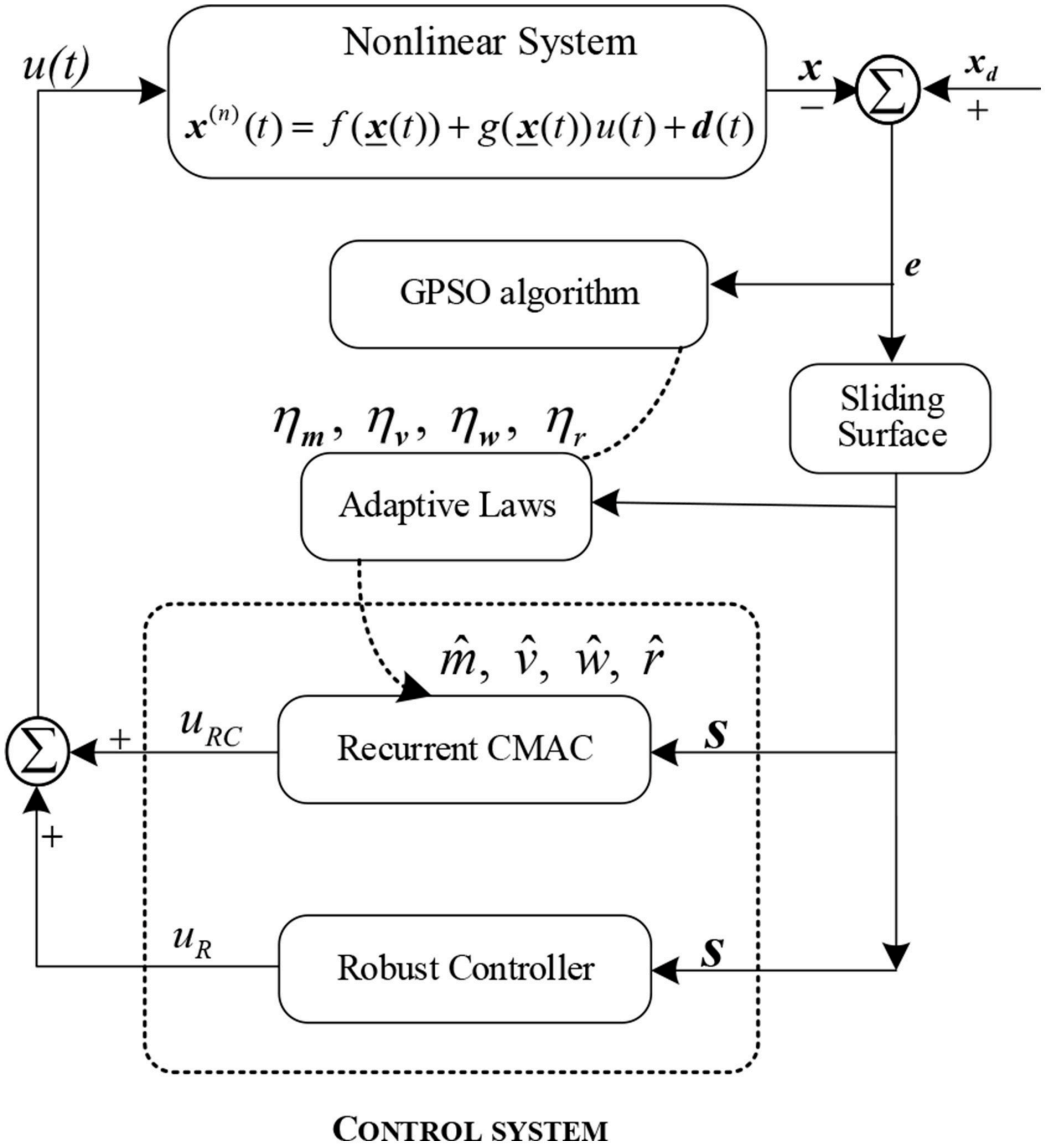
The architecture of the GPSO-RARC control system.

The structure of GPSO-RARC control system consists of a sliding surface, a robust adaptive recurrent CMAC where its learning rates can be updated using the GPSO algorithm and a robust compensation controller. Figure 1 shows the block diagram of the RARC feedback control system.

### The Recurrent CMAC Model

**Figure 2:**
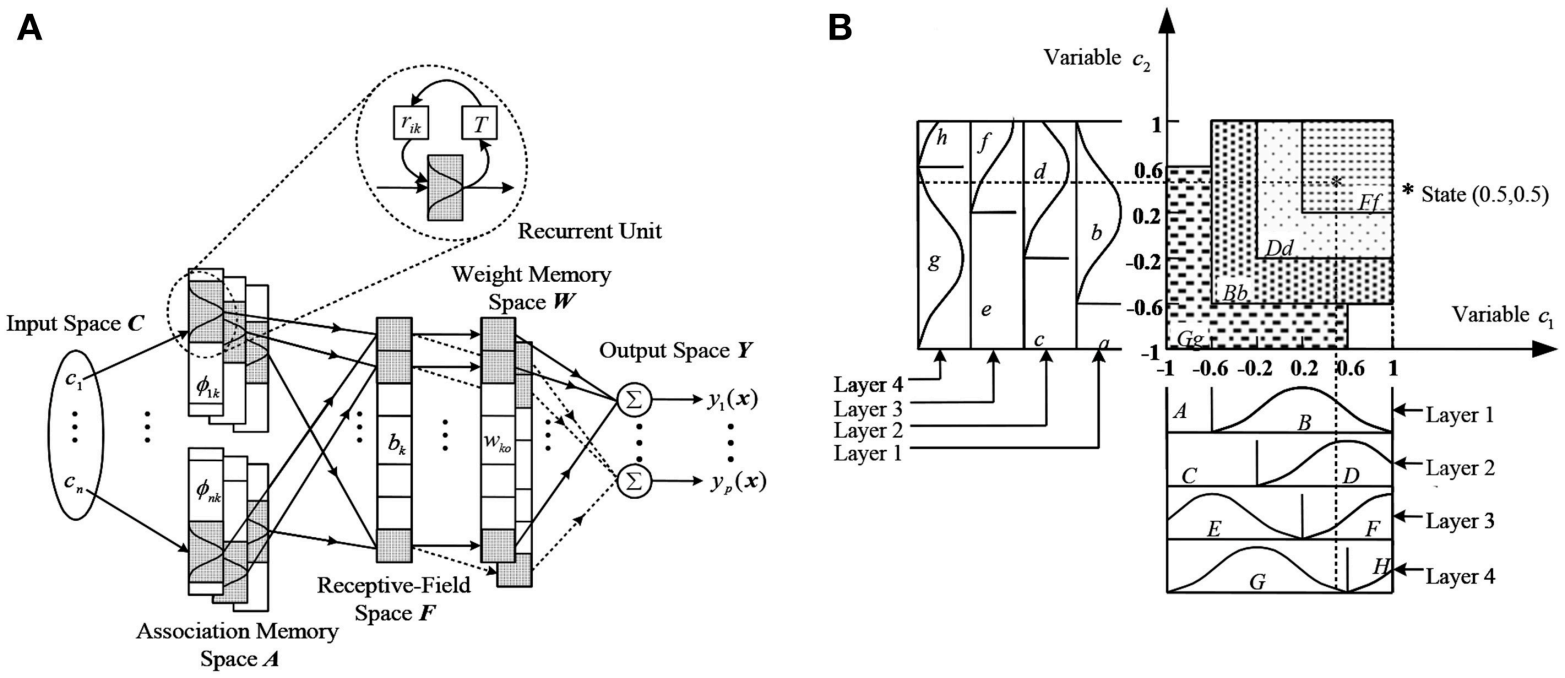
**(A)** Architecture of an RCMAC. **(B)** Organization of a 2-D RCMAC.

Figure 2 Shows an RCMAC model, in which *T* denotes a delay time. The architecture of the RCMAC includes the inputs space, the association memory space with recurrent weights, the receptive-field space, the weight memory space and the outputs space. The following describes the propagation of signals in each space and the basic functions of each space.

1. Input space **C**: which can be described as the $\text{c}={\left[c_{1},\;\cdots \;,c_{i},\;\cdots \;,c_{n_{i}}\right]}^{T}\in {ℜ}^{n_{i}}$, *c*_*i*_ is the *i*th input in layer 1. Based on the specific control space, all variables of the input state *c*_*i*_ can be quantized to discrete regions (namely, an element).
2. Association memory space (Membership function) **A**: usually several elements are accumulated into one block and the number of blocks *n*_*k*_ is usually no less than two. **A** represents an association memory space with *n*_*A*_ (*n*_*A*_ = *n*_*i*_ × *n*_*k*_) components. In the space, each block performs the Gaussian function as a receptive-field basis function, and is described as:
  (6) $$\begin{array}{l} \phi_{ik}=\exp\left[\frac{-{\left(cr_{i}-m_{ik}\right)}^{2}}{v_{ik}^{2}}\right],for\;i=1,2,\ldots ,n_{i},\;k=1,2,\cdots \;,n_{k} \end{array}$$
  in which *ϕ*_*ik*_ denotes the *k*th block of the *i*th input *cr*_*i*_ with the mean *m*_*ik*_ and the variance *v*_*ik*_. In general, the input of this block can be described as follows:
  (7) $$\begin{array}{l} cr_{i}\left(t\right)=c_{i}\left(t\right)+r_{ik}\;\phi_{ik}\left(t-T\right) \end{array}$$
  in which represents the recurrent gain, $\phi_{ik}(t-T)\underline{\underline{\Delta }}\phi_{ikT}$ indicates the value of *ϕ*_*ik*_ through time delay *T*. Obviously, the input of this block includes the memory term *ϕ*_*ikT*_, which saves the previous information about the network and presents dynamic mapping. This is the obvious difference between RCMAC and traditional CMAC. Where the variable *c*_1_ is separated into blocks *A* and, while the variable *c*_2_ is separated into blocks *a* and *b*. Shifting each variable to an element yields different blocks. For example, in Figure 2B the block *C* and *D* for *c*_1_, while the block *c* and *d* for *c*_2_ are obtained by shifting an element. In this space, each block has three adjustable parameters, named *m*_*ik*_, *v*_*ik*_, and *r*_*ik*_.
3. Receptive-field space (Hypercube) : *H* regions composed of blocks (called Aa and Bb) are called receptive-fields. The *k*th multidimensional receptive field function is described as follow:
  (8) $$\begin{array}{l} \begin{array}{l} h_{k}(cr,m_{k},v_{k},r_{k})=\prod_{i\;=\;1}^{n_{i}}\phi_{ik}=\prod_{i=1}^{n_{i}}\exp\left(-{\left(\frac{cr_{i}-m_{ik}}{v_{ik}}\right)}^{2}\right)\\ \text{ for }i=1,2,...,n_{i},\text{ and }k=1,2,...,n_{k} \end{array} \end{array}$$
  in which $\text{c}r={\left[cr_{1},\;cr_{2},\;\cdots \;,\;cr_{n_{i}}\right]}^{T}\in R^{n_{i}}$, ${\text{m}}_{k}={\left[m_{1k},\;m_{2k},\;\cdots \;,\;m_{n_{i}k}\right]}^{T}\in R^{n_{i}}$ and ${\text{v}}_{k}={\left[v_{1k},\;v_{2k},\;\cdots \;,\;v_{n_{i}k}\right]}^{T}\in R^{n_{i}}$. Meanwhile, the multidimensional receptive-field functions can also be expressed in a vector form:
  (9) $$\begin{array}{l} \text{H}={\left[h_{11},\ldots ,h_{1n_{k}},h_{21},\ldots ,h_{2n_{k}},\ldots ,h_{n_{i}1},\ldots ,h_{n_{i}n_{k}}\right]}^{T}\in {ℜ}^{n_{i}n_{k}}\\ ={\left[h_{1},\ldots ,h_{l},\ldots ,h_{n_{l}}\right]}^{T}\in {ℜ}^{n_{l}} \end{array}$$
  in which *h*_*ik*_ is associated with the *i*th layer and *k*th block, the field is activated while the input is in the *k*th receptive-field. At the same time, one or more of the same weights are activated by nearby inputs, and the corresponding blocks export similar outputs. The correlation is a very useful feature of the RCMAC, which is a local generalization.
4. Weight memory (RCMAC output weight) ***W***: in this space, the parameter *w*_*n*_*i*_*n*_*k*__ is the weight which parameterizes the RCMAC mapping(connects to *h*_*ik*_), which can be represented by the following formula:
  (10) $$\begin{array}{l} \text{w}={\left[w_{11},\ldots ,w_{1n_{k}},w_{21},\ldots ,w_{2n_{k}},\ldots ,w_{n_{i}1},\ldots ,w_{n_{i}n_{k}}\right]}^{T}\in {ℜ}^{n_{i}n_{k}}\\ ={\left[w_{1},\ldots ,w_{l},\ldots ,w_{n_{l}}\right]}^{T}\in {ℜ}^{n_{l}} \end{array}$$
  in which *w*_*l*_ is automatically adjusted from the initial value via the online algorithm.
5. Output space ***Y***: The outputs of RCMAC are the sum of the activated receptive field multiplied by the corresponding weight, expressed as:
  (11) $$\begin{array}{l} u_{RCMAC}=y_{o}=\overset{n_{i}}{\underset{i=1}{\sum }}\overset{n_{k}}{\underset{k=1}{\sum }}w_{ik}h_{ik} \end{array}$$
  and the outputs with the RCMAC can be described with the following vector form as:
  (12) $$\begin{array}{l} u_{RCMAC}=\text{y}={\text{w}}^{T}\text{H} \end{array}$$
  Since the recurrent unit of RCMAC contains the past value of the receptive-field basis function, the results of the control network have the features of dynamic characteristics and simple structure. If the time delay T = 0, the system will return to a conventional CMAC mode. Moreover, the RCMAC will be simplified as a recurrent neural network, in case of each block carries only an element, and each input space has only one layer. Therefore, RCMAC is a generalization of recurrent neural networks, but it is more general, faster to learn and more to recall than the latter.

### Adaptive Law for RCMAC Control System

The robust adaptive RCMAC control system includes an adaptive recurrent CMAC and a robust controller which is shown in Figure 1, and output of the system as the following:

(13) $$\begin{array}{l} u\left(t\right)=u_{RARC}=u_{ARCMAC}+u_{R} \end{array}$$

in which *u*_*ARCMAC*_ is the output of the developed adaptive RCMAC and *u*_*R*_ is the output of the robust compensation. *u*_*ARCMAC*_ is the main controller of RCMAC, which is used to approximate the ideal controller in formula (4). The parameters of RCMAC are adjusted online by the adaptive laws. *u*_*R*_ is the robust controller used to efficiently restrain the influence of residual approximation error between the RCMAC and the ideal controller, and guarantees the *L*_2_-stability of the control system.

A sliding surface ***s***(*t*) can be defined as follow:

(14) $$\begin{array}{l} s\left(t\right)=e^{\left(n-1\right)}+K_{1}e^{\left(n-2\right)}+\cdots +K_{n-1}e+K_{n}\int_{0}^{t}e\left(\tau \right)d\tau \end{array}$$

in which $\text{s}\left(t\right)={\left[s_{1}\left(t\right),s_{2}\left(t\right),\cdots s_{m}\left(t\right)\right]}^{T}\in {ℜ}^{m}$, taking the derivative about (14), and substituting with (1) and (13)

(15) $$\begin{array}{l} \dot{s}\left(t\right)=e^{\left(n\right)}+K_{1}e^{\left(n-1\right)}+\cdots +K_{n}\dot{e}=e^{\left(n\right)}+{\underline{K}}^{T}E\\ ={x_{d}}^{\left(n\right)}-f\left(\underline{x}\right)-g\left(\underline{x}\right)u\left(t\right)-d\left(t\right)+{\underline{K}}^{T}E \end{array}$$

then define $L=\frac{1}{2}s^{2}\left(t\right)$ as the cost function, and its derivative is $\dot{L}=s\left(t\right)\dot{s}\left(t\right)\leq 0$ and substituting (13) in it

(16) $$\begin{array}{l} \dot{L}=s(t)\dot{s}(t)=s(t)[x_{d}{\;}^{\left(n\right)}-f(\underline{x})-g(\underline{x})(u_{RCMAC}+u_{R})\\ \;-d(t)+{\underline{K}}^{T}E] \end{array}$$

According to the steepest gradient descent algorithm, the parameters of RCMAC, ${\dot{ŵ}}_{k}$, ${\dot{\hat{m}}}_{ik}$, ${\dot{\hat{v}}}_{ik}$ and ${\dot{\hat{r}}}_{ik}$ can be updated by the tuning laws as below:

(17) $$\begin{array}{l} {\dot{ŵ}}_{k}=-\eta_{w}\frac{\partial s\left(t\right)\;\dot{s}\left(t\right)}{\partial {ŵ}_{k}}=-\eta_{w}\frac{\partial s\left(t\right)\;\dot{s}\left(t\right)}{\partial u_{RCMAC}}\frac{\partial u_{RCMAC}}{\partial {ŵ}_{k}}\\ =\eta_{w}s\left(t\right){gh}_{\;k} \end{array}$$

(18) $$\begin{array}{l} {\dot{\hat{m}}}_{ik}=-\eta_{m}\frac{\partial \;s\left(t\right)\dot{s}\left(t\right)}{\partial u_{RCMAC}}\frac{\partial u_{RCMAC}}{\partial h_{k}}\frac{\partial h_{k}}{\partial \phi_{{\;}_{ik}}}\frac{\partial \phi_{{\;}_{ik}}}{\partial {\hat{m}}_{ik}}\\ \;=\eta_{m}s\left(t\right){gŵ}_{k}h_{k}\frac{2\left(cr_{i}-m_{ik}\right)}{{\hat{v}}_{ik}^{2}} \end{array}$$

(19) $$\begin{array}{l} {\dot{\hat{v}}}_{ik}=-\eta_{v}\frac{\partial \;s\left(t\right)\dot{s}\left(t\right)}{\partial u_{RCMAC}}\frac{\partial u_{RCMAC}}{\partial h_{k}}\frac{\partial h_{k}}{\partial \phi_{{\;}_{ik}}}\frac{\partial \phi_{{\;}_{ik}}}{\partial {\hat{v}}_{ik}}\\ \;=\eta_{v}s\left(t\right){gŵ}_{k}h_{k}\frac{2{\left(cr_{i}-m_{ik}\right)}^{2}}{{\hat{v}}_{ik}^{3}} \end{array}$$

(20) $$\begin{array}{l} {\dot{\hat{r}}}_{ik}=-\eta_{r}\frac{\partial \;s\left(t\right)\dot{s}\left(t\right)}{\partial u_{RCMAC}}\frac{\partial u_{RCMAC}}{\partial h_{k}}\frac{\partial h_{k}}{\partial \phi_{{\;}_{ik}}}\frac{\partial \phi_{{\;}_{ik}}}{\partial {\hat{r}}_{ik}}\\ \;=\eta_{r}s\left(t\right){gŵ}_{k}h_{k}\frac{2\left(cr_{i}-m_{ik}\right)}{{\hat{v}}_{ik}^{2}}\phi_{{\;}_{ikT}} \end{array}$$

where learning-rates η_*w*_, η_*m*_, and η_*r*_ are positive for ${\dot{ŵ}}_{k}$, ${\dot{\hat{m}}}_{ik}$, ${\dot{\hat{v}}}_{ik}$, and ${\dot{\hat{r}}}_{ik}$, respectively.

### Genetic Particle Swarm Optimization (GPSO) Algorithm

Particle swarm optimization algorithm (PSO) is a swarm intelligence algorithm designed by simulating hunting behavior of birds. The PSO moves the individuals in the population to a good region according to the adaptability of the environment. However, instead of using evolutionary operators, each individual fly in the D-dimensional search space at a certain speed and is regarded as a non-volume particle, and dynamically adjusts according to the flight experience of itself and its companions. The *i*th particle is represented as *X*_*i*_ = (*x*_*i*1_, *x*_*i*2_, … *x*_*iD*_), the best position (with the best adaptive value) it has experienced is *P*_*i*_ = (*p*_*i*1_, *p*_*i*2_, …_*p*_*i*_*D*_), also known as *p*_*best*_. The index number of the best position experienced by all particles in the population is denoted by the symbol *g*, namely *P*_*g*_, also known as *g*_*best*_. The velocity of the particle *i* is *V*_*i*_ = (*v*_*i*1_, *v*_*i*2_…, *v*_*iD*_). For each generation, its *d* dimension (1 ≤ *d* ≤ *D*) is changed according to the following equation:

(21) $$\begin{array}{ll} v_{i,d}\left(k+1\right)=\omega \cdot v_{i,d}\left(k\right)+c_{1}\cdot r_{1}\cdot \left(p_{best}-x_{i,d}\right)\\ + & c_{2}\cdot r_{2}\cdot \left(g_{best}-x_{i,d}\right) \end{array}$$

(22) $$\begin{array}{l} x_{i,d}\left(k+1\right)=x_{i,d}\left(k\right)+v_{i,d}\left(k+1\right) \end{array}$$

where *c*_1_ and *c*_2_ are the learning factors, which are also called acceleration constant, ω is the inertia factor, *r*_1_ and *r*_2_ are the uniform random numbers within the range of [0,1]. The right side of the formula (21) consists of three parts. The first part is the inertia or momentum part, which reflects the movement habit of the particle, which means that the particle has a tendency to maintain its previous speed. The second part is the cognition part, which reflects the memory or remembrance of the particle's own historical experience, which represents the tendency of the particle to approach its best position in history. The third part is the social part, which reflects the group history experience of synergy and knowledge sharing between particles.

Particle swarm optimization is simple to calculate and converges quickly, but it lacks mutation ability and is easy to diverge. Genetic algorithm has strong global search ability and high efficiency, but it is prone to premature convergence and poor local search ability. Therefore, a GPSO algorithm is proposed in this paper, which integrates the crossover and mutation operations of the genetic algorithm into the optimization iteration process of particle swarm, and adopts adaptive crossover and adaptive mutation to enhance the ability of the population to jump out of the local optimal solution.

Firstly, the main parameters of PSO are improved in the GPSO algorithm. The linear decreasing method is adopted for inertia weight ω, so that the algorithm can have strong global optimization ability in the early stage of search and detailed local search in the late stage of search. The iterative formula is shown in equation (23):

(23) $$\begin{array}{l} \omega \left(k\right)=\omega_{start}-\left(\omega_{start}-\omega_{end}\right)k/k_{\max} \end{array}$$

where ω_*start*_ is the weight of initial inertia, ω_*end*_ is the weight of terminate inertia; *k* is the current number of iterations; *k*_max_ is the maximum number of iterations.

In order to make the algorithm have a strong global search ability in the early iteration process, it can converge to the global optimal quickly in the later stage, the value of learning factors in this paper is evaluated by asymmetric linear variation, as shown in equations (24) and (25):

(24) $$\begin{array}{l} c_{1}=c_{1s}-\left(c_{1s}-c_{1e}\right)k/k_{\max} \end{array}$$

(25) $$\begin{array}{l} c_{2}=c_{2s}-\left(c_{2s}-c_{2e}\right)k/k_{\max} \end{array}$$

where *c*_1*s*_, *c*_2*s*_ and *c*_1*e*_, *c*_2*e*_ are the initial and terminate iterative value of learning factors of *c*_1_, *c*_2_ respectively.

Secondly, the crossover operation of GA is applied to PSO in the GPSO algorithm. Particles in the population are selected and randomly paired, and then paired particles are crossed with selected probability *p*_*c*_. For cross particles *x*_*i*_ and *x*_*j*_, the calculation process is shown in equations (26) and (27):

(26) $$\begin{array}{l} \{\begin{array}{ll} x_{i}^{k+1} & =\alpha_{1}x_{i}^{k}+(1-\alpha_{1})x_{j}^{k}\\ x_{j}^{k+1} & =(1-\alpha_{1})x_{i}^{k}+\alpha_{1}x_{j}^{k} \end{array} \end{array}$$

(27) $$\begin{array}{l} \{\begin{array}{ll} v_{i}^{k+1} & =\alpha_{2}v_{i}^{k}+(1-\alpha_{2})v_{j}^{k}\\ v_{j}^{k+1} & =(1-\alpha_{2})v_{i}^{k}+\alpha_{2}v_{j}^{k} \end{array} \end{array}$$

where α_1_, α_2_ are two random numbers within the interval [0, 1], and equations (26) and (27) represent the crossover operation of the position and velocity of the paired particles, respectively.

Then, the mutation operation of GA is applied to PSO in GPSO algorithm. The optimal position of each particle varies with the selected probability *p*_*m*_. Assuming that the D-dimensional variable of the individual optimal value *p*_*i*_ is $p_{i}^{d}$, the variation operation of $p_{i}^{d}$ is carried out with the strategy of random perturbation. The variable β applied is subject to the normal distribution with mean value 0 and variance 1, and its mutation formula is shown in (28).

(28) $$\begin{array}{l} p_{i}^{d}=p_{i}^{d}\left(1+0.5\beta \right) \end{array}$$

The selection of crossover probability *p*_*c*_ and mutation probability *p*_*m*_ is one of the important factors affecting the optimization ability of the algorithm. If the *p*_*c*_ is too small, the generation speed of new individuals will slow down during the iteration. If the *p*_*c*_ is too large, the good individuals that have been generated in the population may be damaged. If *p*_*m*_ is too small, then the ability to generate new individuals by mutation operation will be weakened, which is not conducive to maintaining the diversity of the population. If *p*_*m*_ is too large, it is similar to the random search algorithm. Therefore, this paper proposes an adaptive crossover and adaptive mutation strategy to make *p*_*c*_ and *p*_*m*_ automatically adjust according to the evolutionary state of the population.

The rate and mutation probability are defined as shown in equations (29) and (30):

(29) $$\begin{array}{l} p_{c}=\{\begin{array}{ll} \frac{\left(p_{c1}-p_{c2})(f_{\max}-f^{\prime }\right)}{f_{\max}-f_{avg}} & f^{\prime }\geq f_{avg}\\ p_{c1} & f^{\prime }<f_{avg} \end{array} \end{array}$$

(30) $$\begin{array}{l} p_{m}=\{\begin{array}{ll} \frac{\left(p_{m1}-p_{m2})(f_{\max}-f\right)}{f_{\max}-f_{avg}} & f\geq f_{avg}\\ p_{m1} & f<f_{avg} \end{array} \end{array}$$

where *p*_*c*1_, *p*_*c*2_, *p*_*m*1_, and *p*_*m*2_ are constants, *f*′ represents the fitness value corresponding to the better individuals compared with the two individuals with crossover operation; *f* refers to the fitness function value of the mutant operational particle, *f*_*avg*_ refers to the average value of the fitness function value of the entire population at present. From the formula, it can be seen that the probability of crossover operation and mutation operation of individuals whose fitness function value is lower than the population average is relatively high, which ensures the population diversity. At the same time, when *f*_max_ − *f*_*avg*_ decreases, the individual in the population tends to converge to the local optimal solution. Meanwhile, the probability of individual crossover and mutation will increase, which enhances the ability of the population to generate new individuals and urges them to jump out of the local optimal solution.

**Figure 3:**
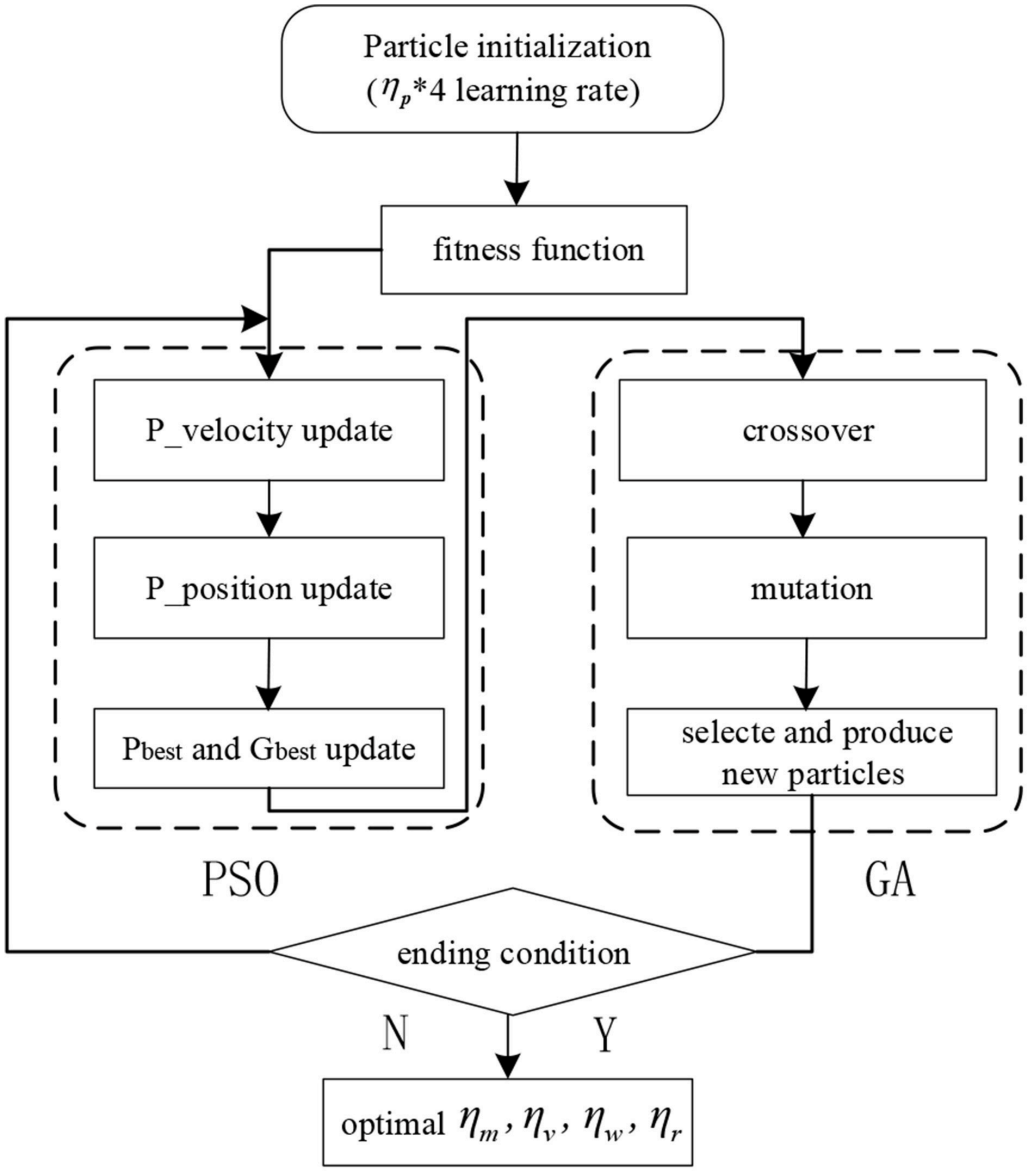
The flowchart of the GPSO algorithm.

Finally, the fitness function $f_{itness}=\overset{m}{\underset{i=1}{\sum }}{\left\|e_{i}\left(t\right)\right\|}^{2}$ is chosen as a cost function, to evaluate the performance of learning rates in the GPSO algorithm. The flowchart of GPSO is shown in Figure 3.

### Robust Compensation Control

There is unavoidably the approximation error between the adaptive recurrent CMAC(ARCMAC) and the ideal controller, an ideal controller can be formulated as the sum of ARCMAC and the approximate error:

(31) $$\begin{array}{l} u^{*}={u_{AR}}_{CMAC}+\varepsilon \left(t\right) \end{array}$$

Substituting (13) in (1), yield:

(32) $$\begin{array}{l} x^{\left(n\right)}=f\left(\underline{\text{x}}\right)+g\left(\underline{\text{x}}\right)\left(u_{ARCMAC}+u_{R}\right)+d\left(t\right) \end{array}$$

using the product of (4) and *g*(*x*) subtract (32), yield:

(33) $$\begin{array}{l} g\left(\underline{\text{x}}\right)\left(u^{*}-u_{ARCMAC}-u_{R}\right)=e^{\left(n\right)}+{\underline{\text{K}}}^{T}E=\dot{s}\left(t\right) \end{array}$$

The robust controller can reduce the influence of the approximation error between the ARCMAC and the ideal controller, thus achieving the tracking performance of *L*_2_. Assuming that ***ε***(*t*) exists and satisfies *L*_2_ bounded, consider the specified *L*_2_ tracking performance (Chen and Lee, 1996):

(34) $$\begin{array}{l} \overset{m}{\underset{i=1}{\sum }}\int_{0}^{\;T}s_{i}^{2}\text{(}t\text{)}dt\;\leq \overset{m}{\underset{i=1}{\sum }}\left[s_{i}^{2}(0)/g_{0i}^{\;}\right]+\overset{m}{\underset{i=1}{\sum }}r_{i}^{2}\int_{0}^{\;T}\varepsilon_{i}^{2}\text{(}t\text{)}dt\; \end{array}$$

Here *r*_*i*_ is a prescribed positive attenuation constant. The following formula describes the design of a robust controller:

(35) $$\begin{array}{l} u_{R}\left(t\right)=\;{\left(2R^{2}\right)}^{-1}\left(R^{2}+I\right)\;s\left(t\right) \end{array}$$

where $R=diag\left(r_{1},r_{2},\cdots \;,r_{m}\right)\in {ℜ}^{m\times m}$ and *I* is the unity matrix, then further state and prove the following theorem.

**Theorem I:** while the *n*th-order MIMO non-linear systems described in (1), the RARC control system is designed as in (13), in which *u*_*RCMAC*_ is shown as (12) with the online parameter learning algorithms (17)-(20), and (35) describe the design of the robust controller. Then the desired *L*_2_ tracking performance in (34) can be achieved for the specified attenuation levels *r*_*i*_, i = 1, 2, ⋯, m.

**Proof:** The following formula gives the Lyapunov function:

(36) $$\begin{array}{l} V\left(s\left(t\right)\right)=\frac{1}{2}s^{T}\left(t\right)s\left(t\right) \end{array}$$

Taking the derivative of the Lyapunov function and using (31), (33) and (35), as below:

(37) $$\begin{array}{l} \begin{array}{ll} \dot{V}(s(t)) & =s^{T}(t)\dot{s}(t)\\ \; & =s^{T}(t)g[\varepsilon (t)-{\left(2R^{2}\right)}^{-1}(R^{2}+I)\;s(t)]\\ \; & =\sum_{i=1}^{m}g_{0i}[s_{i}(t)\varepsilon_{i}(t)-s_{i}^{2}(t)\frac{r_{i}^{2}+1}{2r_{i}^{2}}]\\ \; & =\sum_{i=1}^{m}g_{0i}[s_{i}(t)\varepsilon_{i}(t)-\frac{s_{i}^{2}(t)}{2\;}-\frac{s_{i}^{2}(t)}{2\;r_{i}^{2}})]\\ \; & =\sum_{i=1}^{m}g_{0i}[-\frac{s_{i}^{2}(t)}{2\;}-\frac{1}{2}{\left(\frac{s_{i}(t)}{\;r_{i}}-r_{i}\varepsilon_{i}(t)\right)}^{2}+\frac{r_{i}^{2}\varepsilon_{i}^{2}(t)}{2\;}]\;\\ \; & \leq \sum_{i=1}^{m}g_{0i}[-\frac{s_{i}^{2}(t)}{2\;}+\frac{r_{i}^{2}\varepsilon_{i}^{2}(t)}{2\;}] \end{array} \end{array}$$

Assuming ε_*i*_(*t*) ∈ *L*_2_ [0, *T* ], ∀*T* ∈ [0, ∞), taking the integration of the above equation from *t* = 0 to t= *T*, yields:

(38) $$\begin{array}{l} V\text{(}T\text{)}-V\text{(0)}\leq \overset{m}{\underset{i=1}{\sum }}g_{0i}\;\left[\;-\frac{1}{2}\int_{0}^{\;T}s_{i}^{2}\text{(}t\text{)}dt+\frac{r_{i}^{2}}{2}\int_{0}^{\;T}\varepsilon_{i}^{2}\text{(}t\text{)}dt\;\right]\; \end{array}$$

Since *V*(*T*) ≥ 0, the following inequality can be derived from (38):

(39) $$\begin{array}{l} \frac{1}{2}\overset{m}{\underset{i=1}{\sum }}g_{0i}\int_{\;0}^{\;T}s_{i}^{2}\left(t\right)\;dt\leq \;V\left(0\right)+\frac{1}{2}\overset{m}{\underset{i=1}{\sum }}g_{0i}r_{i}^{2}\int_{\;0}^{\;T}\varepsilon_{i}^{2}\left(t\right)\;dt\; \end{array}$$

using (36), the above inequality is equivalent to the following:

(40) $$\begin{array}{l} \overset{m}{\underset{i=1}{\sum }}\int_{0}^{\;T}s_{i}^{2}\text{(}t\text{)}dt\;\leq \;\overset{m}{\underset{i=1}{\sum }}\left[s_{i}^{2}\left(0\right)/g_{0i}\right]+\overset{m}{\underset{i=1}{\sum }}r_{i}^{2}\int_{0}^{\;T}\varepsilon_{i}^{2}\text{(}t\text{)}dt\; \end{array}$$

and the proof is completed.

Moreover, in (24), in the case of $\int_{0}^{\;T}\varepsilon_{i}^{2}\text{(}t\text{)}dt<\infty$ then $\int_{\;0}^{\;T}s_{i}^{2}\left(t\right)\;dt<\infty$ for all *T*, so the *L*_2_ stability of the closed-loop system is guaranteed.

## Simulation Results

### Single Inverted Pendulum System

**Figure 4:**
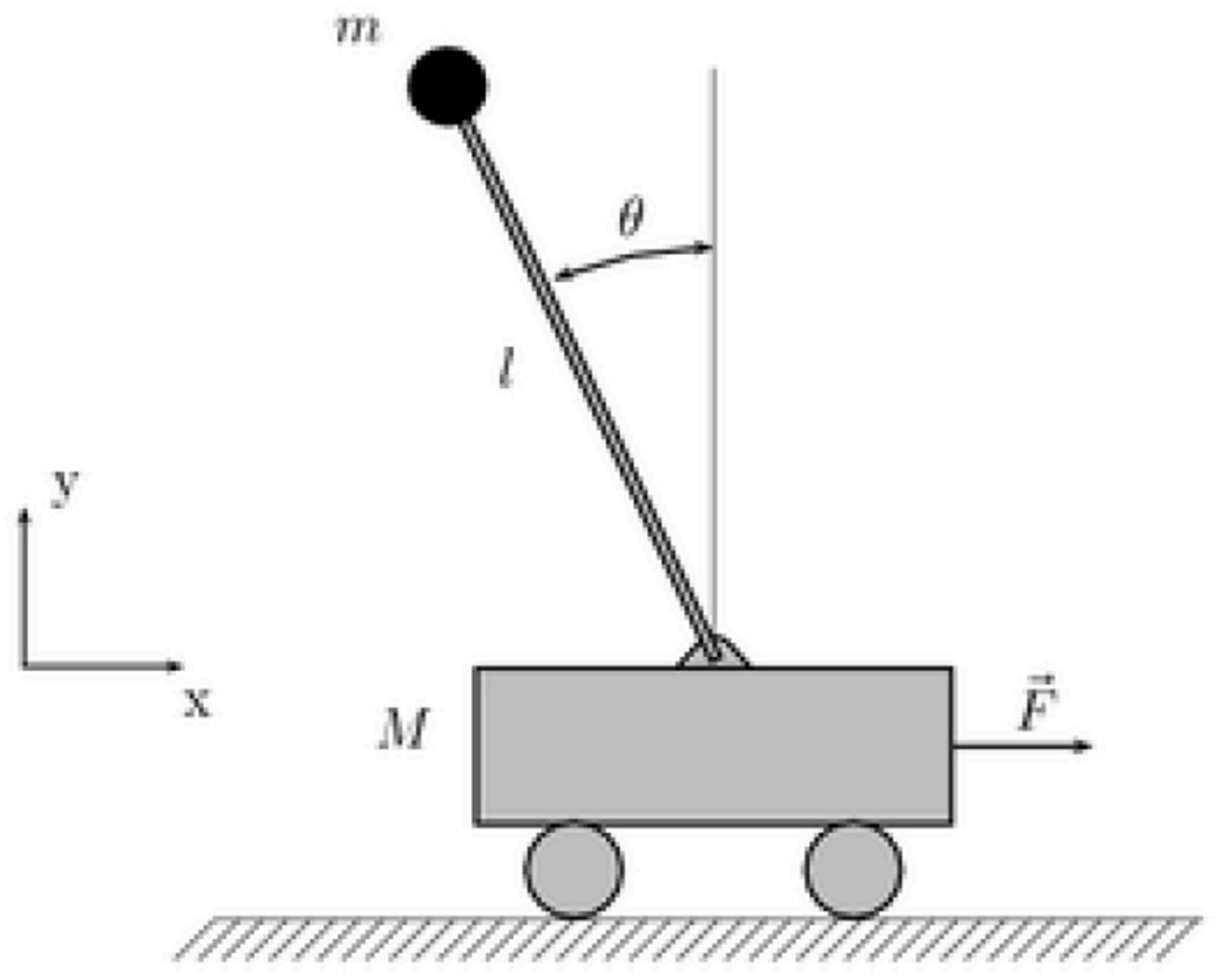
Single inverted pendulum system.

There is the single inverted pendulum system on the vehicle, as shown in Figure 4, and its dynamic equation is as follows (Mori et al., 1976):

(41) $$\begin{array}{l} \begin{array}{c} {ẋ}_{1}=x_{2}\\ {ẋ}_{2}=f\left(\text{x}\right)+g\left(\text{x}\right)u \end{array} \end{array}$$

where, $f\left(\text{x}\right)=\frac{g\sin x_{1}-ml{x_{2}}^{2}\cos x_{1}\sin x_{1}/\left(m_{c}+m\right)}{l\left(4/3-m\cos^{2}x_{1}/\left(m_{c}+m\right)\right)}$, $g\left(\text{x}\right)=\frac{\cos x_{1}/\left(m_{c}+m\right)}{l\left(4/3-m\cos^{2}x_{1}/\left(m_{c}+m\right)\right)}$, *x*_1_ and *x*_2_ are the angle and angular velocity of the pendulum, respectively, *g* = 9.8*m*/*s*^2^ is the acceleration of gravity, *m*_*c*_ = 1*kg* is the mass of the car, is the mass of the pendulum, *l* = 0.5*m* is half the length of the pendulum, *u* is the control input.

The tracking reference signals are *x*_*d*_(*t*) = 0.1*sin*(*t*), the initial conditions for this system are set as *x*(0) = π/60, ẋ(0) = 0, the robust compensation *R* = 0.05, the *k*_1_ = 3, *k*_2_ = 1 then the inputs are $s\left(t\right)=3e+ė+\int_{0}^{t}edt$ and ṡ(*t*) = ë + 3ė + *e*.

**Figure 5:**
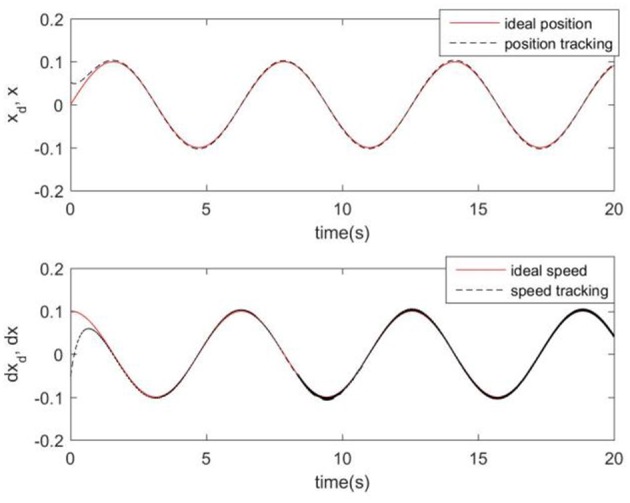
Simulated pendulum systems on angle and angular speed based on RCMAC.

**Figure 6:**
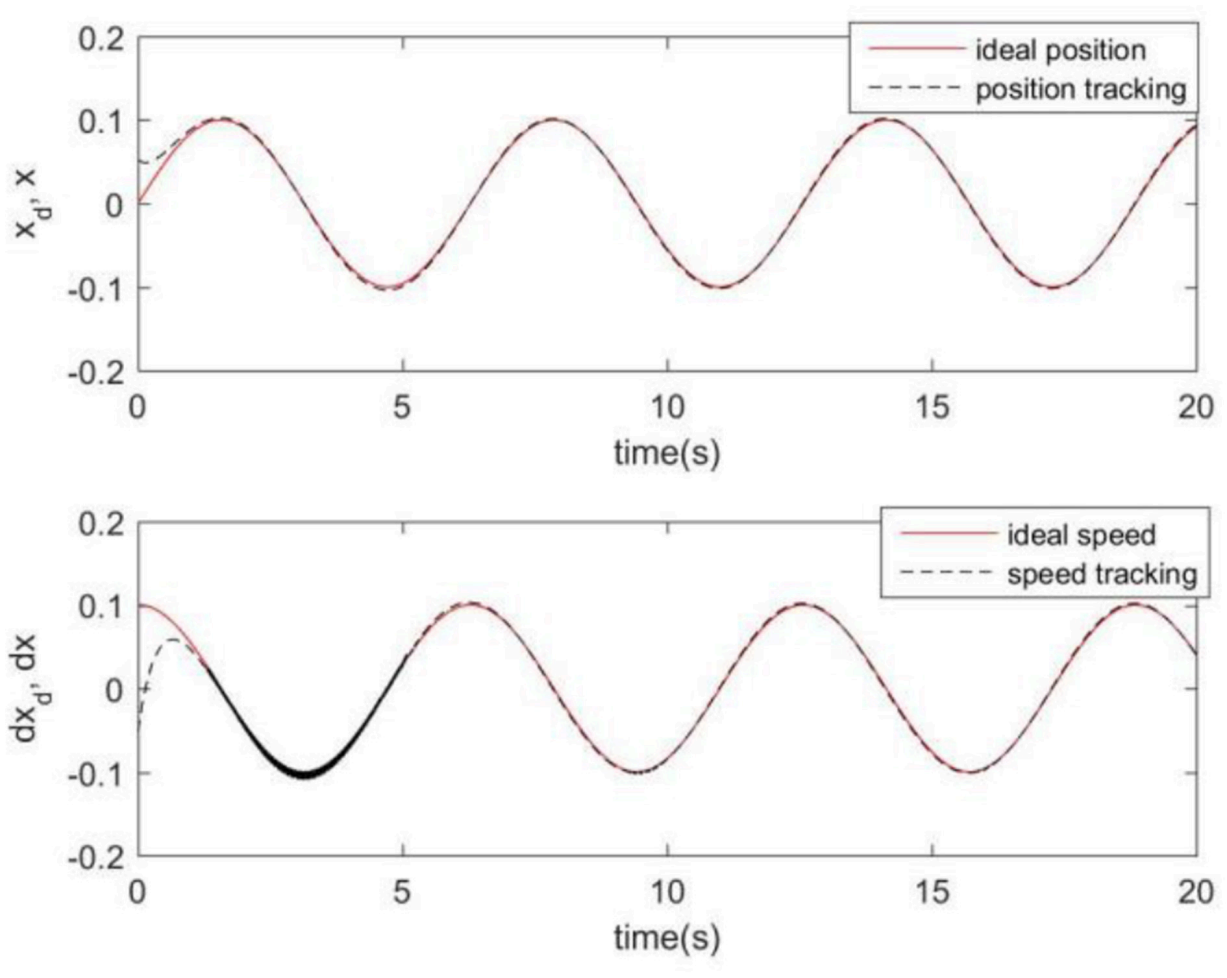
Simulated pendulum systems on angle and angular speed based on RARC_PSO.

**Figure 7:**
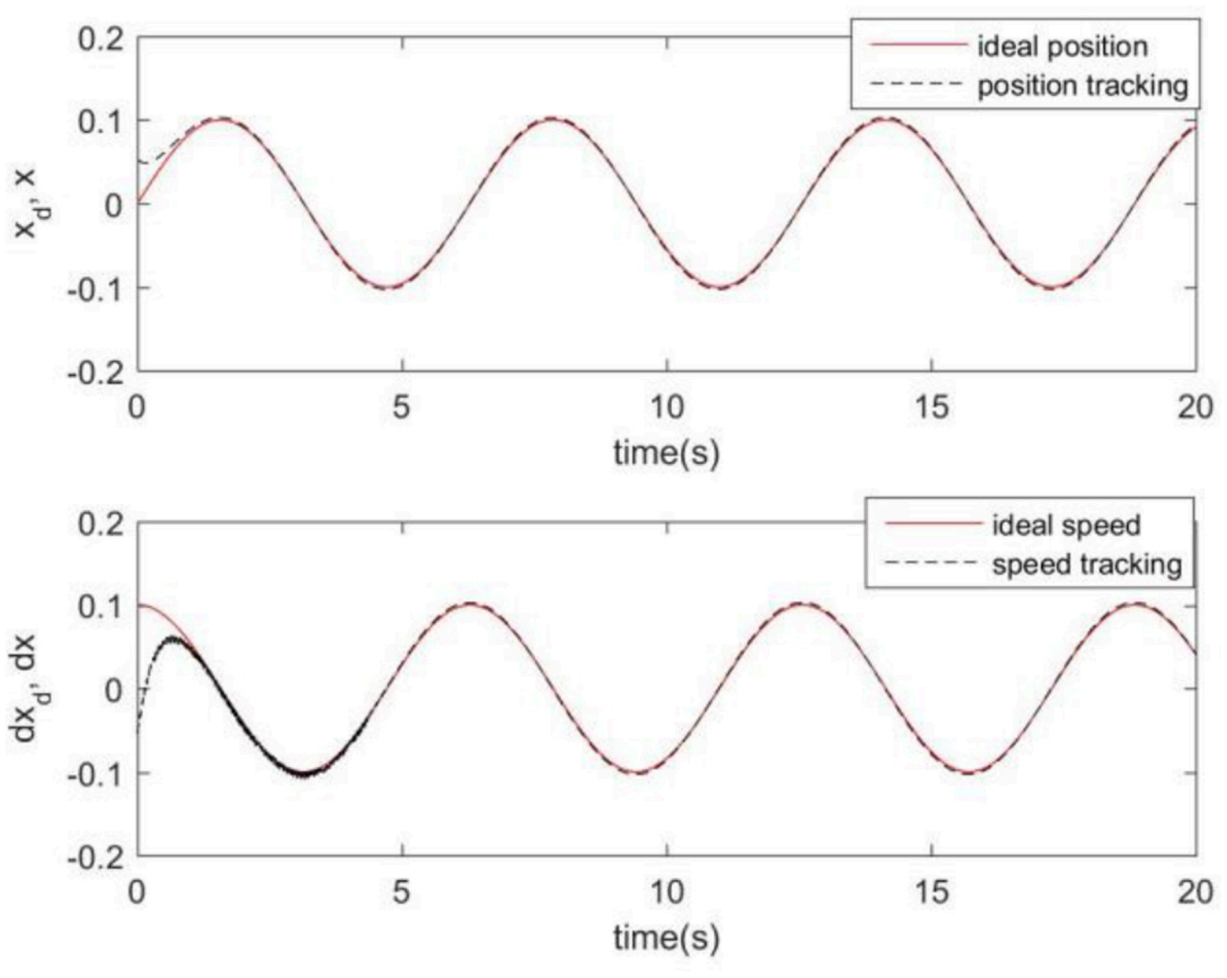
Simulated pendulum systems on angle and angular speed based on RARC_GA.

**Figure 8:**
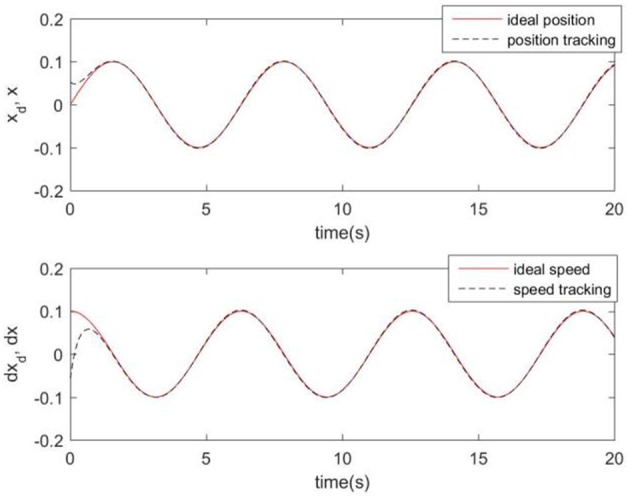
Simulated pendulum systems on angle and angular speed based on RARC_GPSO.

**Figure 9:**
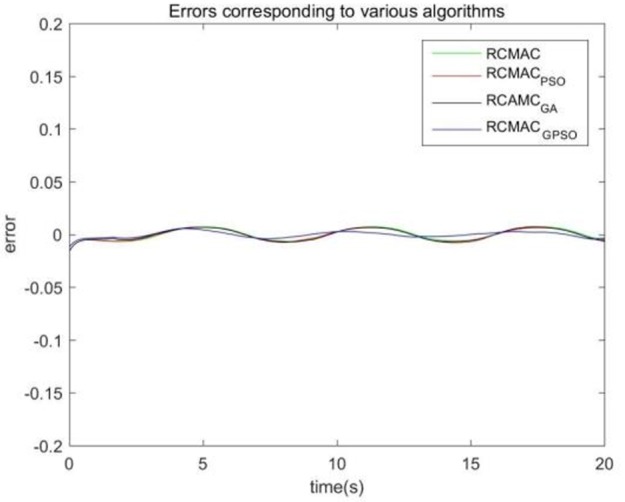
Tracking error for angle on four schemes.

Considering the practical application, the off-line training time of the three optimization algorithms is set to 2 s, then the learning rate obtained by off-line training is taken as the initial value of the learning rate parameter of the system controller. For comparison, the original RCMAC control system, the RARC control system based on GA algorithm, the RARC control system based on PSO algorithm, and the RARC control system based on GPSO algorithm are applied to this single inverted pendulum system. Their simulation results are shown in Figures 5–**9**. The state responses *x*(*t*) and ẋ(*t*) of normal RCMAC and RARC based on PSO (RARC_ PSO) are shown in Figures 5, 6, respectively and state responses of RARC based on GA (RARC_GA) and RARC based on GPSO (RARC_GPSO) are plotted in Figures 7, 8, respectively. Moreover, the tracking errors of various algorithms are depicted in Figure 9, and the Root Mean Square Error (RMSE) are presented in Table 1, respectively. Eventually, the simulation results show the proposed RARC_GPSO control system can effectively achieve favorable control for the single inverted pendulum system and can get better tracking performance than others, especially in the tracking of the angular speed.

**Table 1:** Initial of learning rate and RMSE.

**Algorithms**	**Lr_m**	**Lr_v**	**Lr_w**	**Lr_r**	**Rmse**
RCMAC	0.2	0.3	0.5	0.2	0.0158
RARC_ PSO	0.073	0.059	0.689	0.208	0.0153
RARC_GA	0.021	0.249	0.094	0.519	0.0150
RARC_GPSO	0.050	0.889	0.722	0.291	0.0056

### Two Link Robot Manipulator System

The controlled object is an n-joint robot manipulator, and its non-linear dynamic equation is (Lewis et al., 2003):

(42) $$\begin{array}{l} M\left(x\right)ẍ+V\left(x,ẋ\right)ẋ+G\left(x\right)+F\left(ẋ\right)+\tau_{d}=\tau \end{array}$$

where *M*(*x*) ∈ *R*^*n*×*n*^ indicates inertia matrix which is the symmetrical positive definite, *V*(*x*, ẋ) ∈ *R*^*n*^ means the term of centrifugal force and Coriolis force, Ṁ(*x*) − 2*V*(*x*, ẋ) is the oblique symmetric matrix, *G*(*x*) ∈ *R*^*n*^ is the gravity, *F*(ẋ) ∈ *R*^*n*^ is the term of friction, $\tau_{d}\left(t\right)\in R^{n}$ is an unknown external disturbance, τ(*t*) ∈ *R*^*n*^ is the joint torque vector applied by the actuator, and *x* ∈ *R*^*n*^ represents the vector of the joint variable.

**Figure 10:**
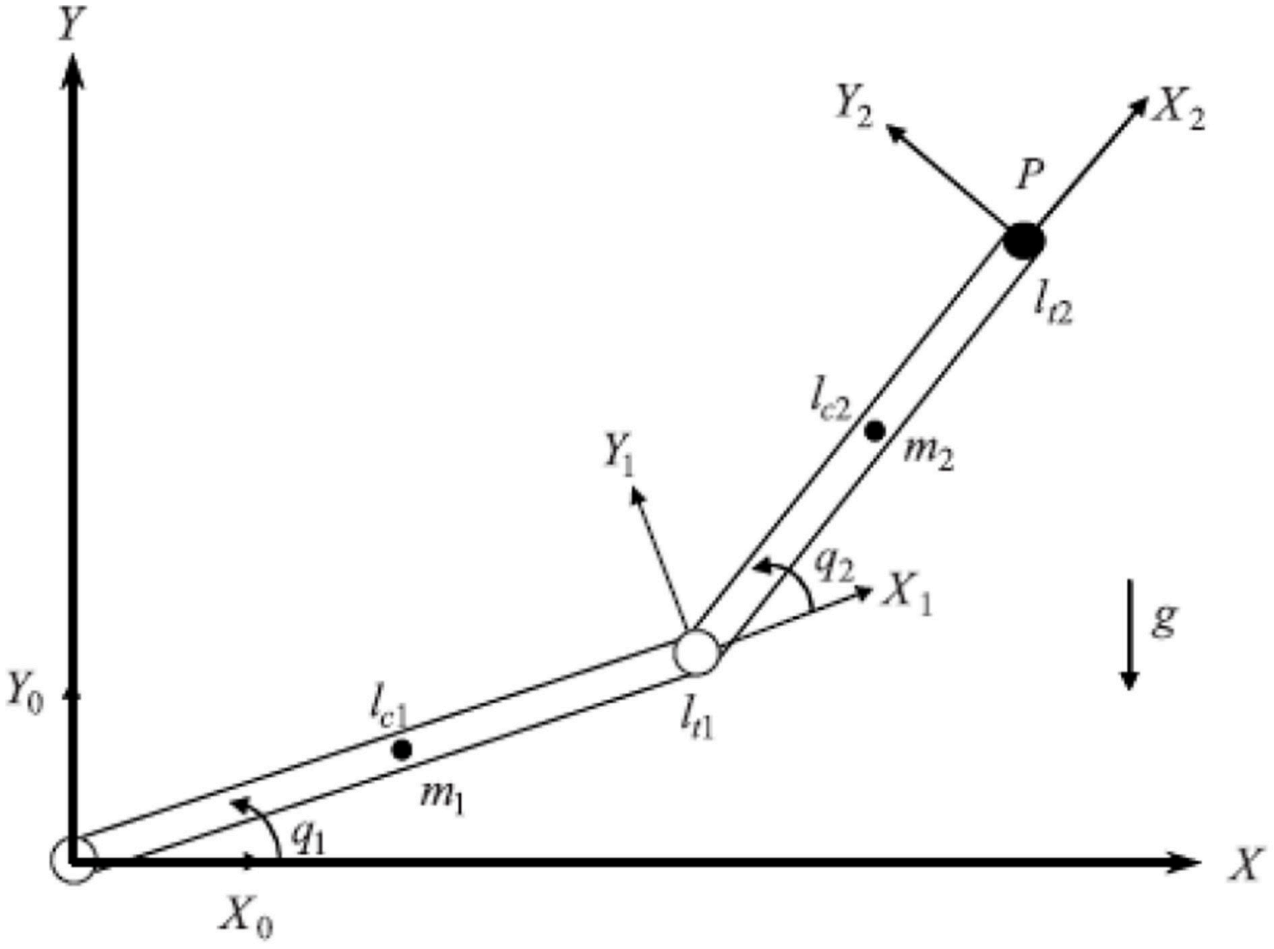
Two-link robot manipulator's architecture.

A two-joint system is presented as follow, as shown in Figure 10, and the similar design process can be extended to any n-joint system. The specific system parameters of the two joint manipulators are described as below:

(43) $$\begin{array}{l} M\left(x\right)\\ =\left[\begin{array}{cc} {l_{2}}^{2}m_{2}+{l_{1}}^{2}\left(m_{1}+m_{2}\right)+2l_{1}l_{2}m_{2}\cos\left(x_{2}\right) & {l_{2}}^{2}m_{2}+l_{1}l_{2}m_{2}\cos\left(x_{2}\right)\\ {l_{2}}^{2}m_{2}+l_{1}l_{2}m_{2}\cos\left(x_{2}\right) & l_{2}m_{2} \end{array}\right] \end{array}$$

(44) $$\begin{array}{l} V\left(x,ẋ\right)=\left[\begin{array}{cc} -l_{1}l_{2}m_{2}{ẋ}_{2}\sin\left(x_{2}\right) & -l_{1}l_{2}m_{2}\left({ẋ}_{1}+{ẋ}_{2}\right)\sin\left(x_{2}\right)\\ m_{2}l_{1}l_{2}\sin\left(x_{2}\right) & 0 \end{array}\right] \end{array}$$

(45) $$\begin{array}{l} G\left(x\right)=\left[\begin{array}{c} \left(m_{1}+m_{2}\right)l_{1}g\cos\left(x_{1}\right)+l_{2}m_{2}\cos\left(x_{1}+x_{2}\right)\\ m_{2}l_{2}g\cos\left(x_{1}+x_{2}\right) \end{array}\right] \end{array}$$

where *x*_1_, *x*_2_, *m*_1_, *m*_2_ and *l*_1_, *l*_2_ are the angle, mass and length of joint 1 and 2, respectively. *g* means the gravity acceleration.

In addition, the dynamics of the manipulator also includes the non-linear viscous and dynamic friction terms of *F*(ẋ) and the unknown disturbance τ_*d*_, as follow:

(46) $$\begin{array}{l} \{\begin{array}{ll} l_{1} & =1.\text{0 m }l_{2}\text{=1.0 m }\\ m_{1} & =\text{0.8 kg }m_{2}\text{=2.3 kg }\\ g & =9{\text{.8 m/s}}^{2}\;\\ F(\dot{x}) & =0.02sgn(\dot{x})\;\\ \tau_{d} & =[\begin{array}{ll} 0.2\sin(t) & 0.2\sin(t \end{array})] \end{array} \end{array}$$

The initial state of the system is ${\left[x_{1d},\;{ẋ}_{1d},\;x_{2d},\;{ẋ}_{2d}\right]}^{T}={\left[0.09\;0\;-0.09\;0\right]}^{T}$, and the expected trajectory are represented as:

(47) $$\begin{array}{l} x_{1d}\left(t\right)=\sin t\;x_{2d}\left(t\right)=\sin t \end{array}$$

in the robust compensation *R* = 0.5*I, *k*_1_ = 3 and *k*_2_ = 1.

**Figure 11:**
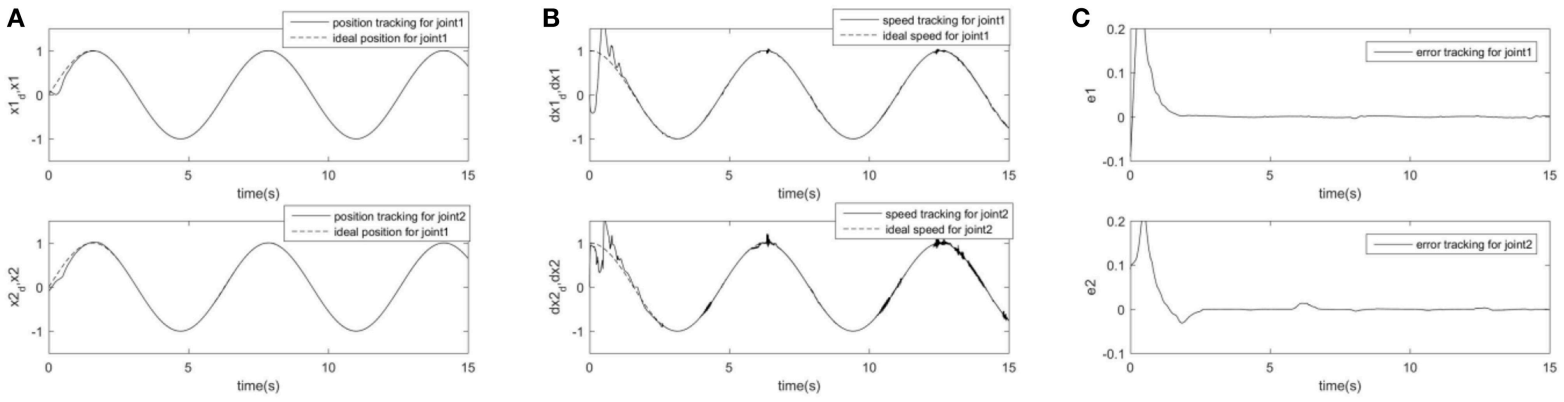
Simulated results of a two-joint manipulator on RCMAC. **(A)** Position tracking for link1 and link2; **(B)** Speed tracking for link1 and link2; **(C)** Tracking error for link1 and link2.

**Figure 12:**
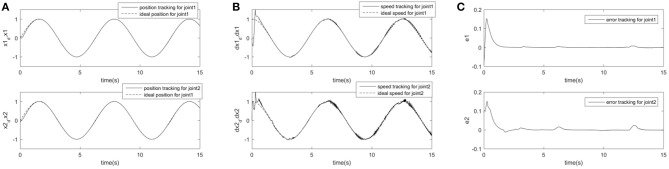
Simulated results of a two-joint manipulator on RARC_GA. **(A)** Position tracking for link1 and link2; **(B)** Speed tracking for link1 and link2; **(C)** Tracking error for link1 and link2.

**Figure 13:**
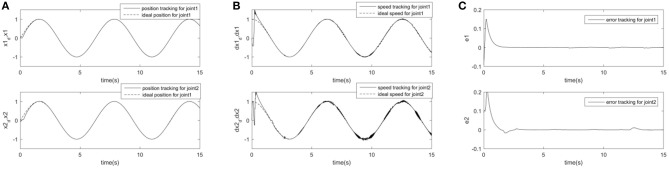
Simulated results of a two-joint manipulator on RARC_PSO. **(A)** Position tracking for link1 and link2; **(B)** Speed tracking for link1 and link2; **(C)** Tracking error for link1 and link2.

**Figure 14:**
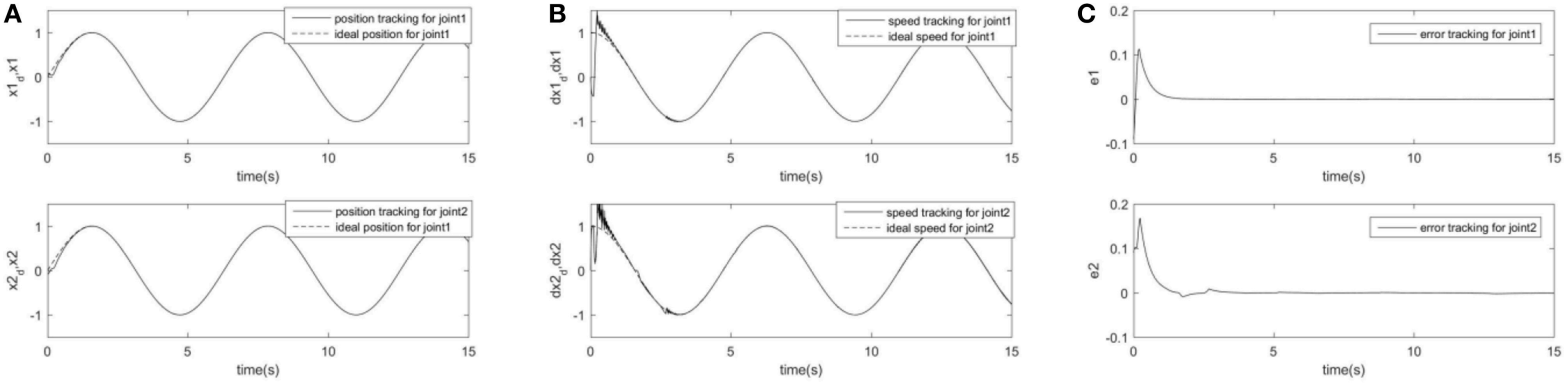
Simulated results of a two-joint manipulator on RARC_GPSO. **(A)** Position tracking for link1 and link2; **(B)** Speed tracking for link1 and link2; **(C)** Tracking error for link1 and link2.

In order to further prove the superiority and robustness of RARC_GPSO control system, the other three neural network control schemes (normal RCMAC NN, RARC_PSO NN and RARC_GA NN) are adopted to compare the position and velocity tracking of the manipulator's joints, as shown in Figures 11–13. The control performance of the robust adaptive RCMAC control system based on GPSO in the two-joint manipulator is shown in Figure 14. It is obvious that the performance of RARC_GPSO is better than that of the other three control methods in the velocity tracking and position tracking, and it has the best speed of error convergence. Table 2 represents RMSE of the four controllers designed above, which reconfirm that the robust adaptive RARC_GPSO is more excellent than the others in robot manipulator control.

**Table 2:** Initial of learning rate and RMSE.

**Algorithms**	**Lr_m1**	**Lr_v1**	**Lr_w1**	**Lr_r1**	**Lr_m2**	**Lr_v2**	**Lr_w2**	**Lr_r2**	**Rmse1**	**Rmse2**
RCMAC	0.1	0.1	0.5	0.1	0.1	0.1	0.5	0.1	0.0442	0.0369
RARC_PSO	0.1639	0.575	0.583	0.032	0.233	0.439	0.544	0.137	0.0194	0.0308
RARC_GA	0.459	0.563	0.456	0.044	0.471	0.586	0.492	0.087	0.0174	0.0279
RARC_GPSO	0.429	0.838	0.600	0.085	0.613	0.704	0.770	0.174	0.0145	0.0185

## Conclusion

A robust adaptive RCMAC control system has been successfully proposed for non-linear MIMO systems in this paper. The main findings of this study are the development of a GPSO-based RCMAC with the adaptive law for updating parameters, and the learning rates can be optimized to best value based on the GPSO algorithm. The control system includes an ARCMAC which is developed to simulate the ideal controller, and a robust controller which is designed to compensate for the difference between ARCMAC and ideal controller. In this design, the optimal learning rate and adaptive learning algorithm of controller parameters are derived, and the *L*_2_-stability of the system is proved by Lyapunov function. Furthermore, the simulation results also prove the effectiveness of the control system. The GPSO-based RCMAC has dynamic characteristics because it considers the past value of received field basis function in associative memory space, so it has outstanding performance in general motion control and trajectory tracking. If the control scheme is applied to classification, the effect is not very good mainly because each sample in the classification is not necessarily linked. The next research plan will refer to the framework of fuzzy theory in the control of nonlinear systems (Zhong et al., 2019), and constantly improve the algorithm to make it universal.

## Author Contributions

JG designed the experiment and drafted the manuscript. SH and SK performed the experiment. YZ processed the data. YS and C-ML modified the paper. All authors read and approved the final manuscript.

### Conflict of Interest Statement

The authors declare that the research was conducted in the absence of any commercial or financial relationships that could be construed as a potential conflict of interest.
